# Mesenchymal stem cell 3D encapsulation technologies for biomimetic microenvironment in tissue regeneration

**DOI:** 10.1186/s13287-018-1130-8

**Published:** 2019-02-07

**Authors:** Hyerim Kim, Chaewon Bae, Yun-Min Kook, Won-Gun Koh, Kangwon Lee, Min Hee Park

**Affiliations:** 1 Program in Nanoscience and Technology, Graduate School of Convergence Science and Technology, Seoul National University, Seoul, Republic of Korea; 2 Department of Chemical and Biomolecular Engineering, Yonsei University, Seoul, Republic of Korea; 3 Advanced Institutes of Convergence Technology, Suwon, Republic of Korea; 4 Center for Convergence Bioceramic Materials, Korea Institute of Ceramic Engineering and Technology, Cheongju, Republic of Korea

**Keywords:** Mesenchymal stem cells, 3D encapsulation, Advanced technologies, Hydrogel, Tissue regeneration

## Abstract

Mesenchymal stem cell (MSC) encapsulation technique has long been emerged in tissue engineering as it plays an important role in implantation of stem cells to regenerate a damaged tissue. MSC encapsulation provides a mimic of a three-dimensional (3D) in vivo environment to maintain cell viability and to induce the stem cell differentiation which regulates MSC fate into multi-lineages. Moreover, the 3D matrix surrounding MSCs protects them from the human innate immune system and allows the diffusion of biomolecules such as oxygen, cytokines, and growth factors. Therefore, many technologies are being developed to create MSC encapsulation platforms with diverse materials, shapes, and sizes. The conditions of the platform are determined by the targeted tissue and translation method. This review introduces several details of MSC encapsulation technologies such as micromolding, electrostatic droplet extrusion, microfluidics, and bioprinting and their application for tissue regeneration. Lastly, some of the challenges and future direction of MSC encapsulation technologies as a cell therapy-based tissue regeneration method will be discussed.

## Background

To treat tissue and organ injuries, cell-based therapy through the transplantation of stem cells into the damaged site to generate new tissue has been the novel approach for tissue regeneration. Among the various types of stem cells, mesenchymal stem cells (MSCs) are commonly used for cell therapy because of many advantages, such as their capacity for self-renewal and differentiation into multi-lineages without ethical issue. Moreover, they have a low risk of teratoma development and low immunogenicity [1, 2]. MSCs can be derived from many types of tissues, for example, the bone marrow, adipose tissue, umbilical cord blood, placenta, lung, liver, and skin [3, 4]. Thus, MSCs have been observed to differentiate into many types of tissue including bone, cartilage, muscle, fat, tendon, ligament, and other connective tissues [5]. Moreover, MSCs secrete various cytokines and growth factors such as interleukin-2 (IL-2), interleukin-8 (IL-8), monocyte chemotactic protein-1 (MCP-1), stromal-derived factor-1 (SDF-1), vascular endothelial growth factor (VEGF), and transforming growth factor-beta (TGF-β) which regulate the immune system as well as many intercellular signaling pathways [6, 7]. These secreted bioactive molecules stimulate organotypic cells, enhancing their activities, and reduce fibrosis and apoptosis [8]. Therefore, MSCs are not only capable of differentiation, but also of affecting various reactions and signaling pathways in the human body.

To deliver MSCs and maintain their advantages (i.e., their capacity for viability and differentiation) in damaged tissues, it is essential to mimic the in vivo microenvironment through three-dimensional (3D) construction and as such retain the cell’s various effects under the 3D environment, such as their phenotype, adhesion, metabolism, and response signal to soluble factors [9]. In fact, cells show different physiological and morphological results in two-dimensional (2D) and 3D environments [10]. In particular, MSCs have better osteogenic [11], adipogenic [12], and hepatic [13, 14] differentiation behavior in the 3D environment. Moreover, MSCs show improved differentiation when they are co-cultured with other types of cells such as human umbilical vein endothelial cells (HUVECs) [15], osteoblasts [16], and hematopoietic stem/progenitor cells (HSPCs) [17] compared with those cultured alone in the 3D environment. This is because MSCs interact with other cells differently in the 3D environment compare with the 2D monolayer environment, which enhances the co-culture effect and results in increased cell expansion and tissue regeneration.

The encapsulation of MSCs, by entrapping the viable cells in a 3D semi-permeable hydrogel matrix, is one of the simple methods to introduce a 3D environment. The cell encapsulation is accomplished through the solidification of a cell-suspended liquid material [18]. The 3D cell-encapsulating matrix should safely deliver the MSCs and maintain their viability and function in vitro and in vivo to ultimately have therapeutic potential. Successfully encapsulated MSCs can then differentiate into the targeted lineages, for example, tendon [19], intervertebral disk [20], bone [21], and articular cartilage [22]. Accordingly, the encapsulation of MSCs into a 3D matrix is a very efficient and effective method in tissue regeneration, and many 3D encapsulation technologies have been developed as powerful tools for regenerative medicine. A variety of cell encapsulation technologies can produce 3D matrices of various shapes and sizes, which affect the viability and differentiation of MSCs. For effective tissue regeneration, the shape and size of the 3D matrix must be determined selectively, depending on the property of the target tissue and the materials used (Table 1). Therefore, it is crucial to understand the principles and processes of the various MSC encapsulation technologies in order to select the most efficient and effective one for the intended purpose.

**Table 1:** Summary of encapsulation technologies with diverse materials and MSC types for different target tissues

Technologies		Benefits and limitations	Materials	MSC type	Target tissue	Reference
Micromolding		Benefits: • Controlled shape • Controlled size Limitations: • Batch process	Fibrin	Human bone marrow-derived stem cell	Blood vessel	[36]
			Alginate	Bone marrow-derived stem cell	Non-specific	[37]
			Polyethylene glycol (PEG)-based hydrogel	Human mesenchymal stem cells	Non-specific	[38]
Electrostatic droplet extrusion		Benefits: • Controlled droplet size • Uniform droplet size Limitations: • Materials constraints	Alginate	Rat adipose-derived stem cell	Non-specific	[50]
			Alginate	Human adipose-derived stem cell	Non-specific	[51]
			Alginate-lyase	Rat adipose-derived stem cell	Bone	[85]
Microfluidics	Droplet	Benefits: • Controlled monodispersity • Controlled dimensions and shape Limitations: • Non-scalable	Gelatin norbornene (GelNB) and PEG	Human bone marrow-derived stem cell	Hyaline cartilage	[60]
			Gelatin methacryloyl (GelMA)	Rat bone marrow-derived stem cell	Bone	[61]
			Alginate/RGD-alginate	Human bone marrow-derived stem cell	Bone	[87]
			RGD-alginate	Human periodontal ligament stem cell/gingival mesenchymal stem cell	Cartilage	[84]
	Microfiber	Benefits: • Homogeneous • Continuous Limitations: • Flow friction • Clogging in microchannels	Alginate	Mouse bone marrow-derived stem cell	Blood vessel	[65]
Bioprinting	Inkjet bioprinting	Benefits: • Low cost • High-throughput Limitations: • Nozzle clogging • Non-uniform droplet size	PEG-GelMA	Human bone marrow-derived stem cell	Cartilage/bone	[69]
			Type I collagen- and chitosan-agarose blends	Human bone marrow-derived stem cell	Adipose/bone	[70]
			Fibrin-collagen	Human amniotic fluid-derived stem cell and bone marrow-derived stem cell	Skin	[90]
			Agarose-collagen	Human bone marrow-derived stem cell	Bone	[86]
	Extrusion bioprinting	Benefits: • High cell density • Wide range of viscous materials Limitations: • Slow speed • Viscous liquid only	Methacrylamide gelatin	Rat bone marrow-derived stem cell	Bone	[73]
			Polylactic acid (PLA)/GelMA	Rat bone marrow-derived stem cell	Bone	[74]
			Cellulose and alginate	Human bone marrow-derived stem cell	Cartilage	[83]
			Skin-derived ECM	Human adipose-derived mesenchymal stem cell	Skin	[89]
	Laser-assisted bioprinting	Benefits: • High cell viability • Various bioink available Limitations: • High cost	Polyester urethane urea (PEUU)	Human bone marrow-derived stem cell	Cardiac	[79]
			Plasma-alginate	Porcine bone marrow-derived stem cell	Cartilage/bone	[80]

In this review, we discuss the various MSC encapsulation technologies, from the old methods to the recently developed ones. We also describe several applicable tissue regeneration strategies with the introduced encapsulation technologies. Finally, the challenges of MSC encapsulation and its future direction for tissue regeneration will be presented.

## MSC encapsulation technology

**Fig. 1:**
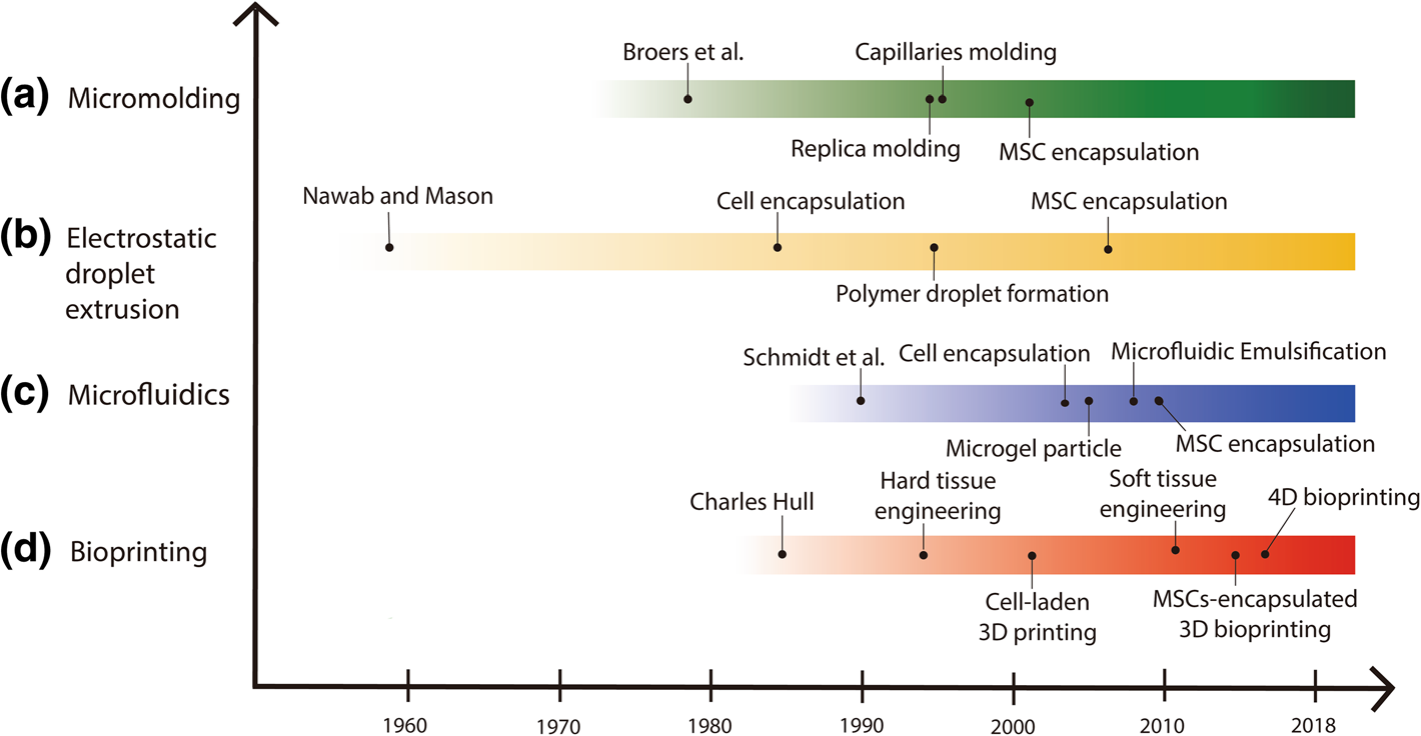
Technical history and principal description of the technologies developed to achieve cell encapsulation, by year. **a** Micromolding was used in a variety of fields, but not for cell encapsulation until the early 1980s. Lithography based on micromolding was founded by Broers et al. [92] whereas techniques using replica [93] and capillary molding [94] were developed in 1996, and MSC encapsulation began in 2002. **b** Nawab and Mason suggested liquid droplets under electrostatic fields, which formed the principle of electrostatic droplet extrusion in 1958 [95]. For cell encapsulation using this technology, Goosen et al. proposed cell immobilization within a semipermeable membrane [96]. Moreover, Bugarski et al. proved the mechanism of polymer droplet formation with electrostatic droplet extrusion in 1994 [42]. Finally, MSC encapsulation was conducted in the late 2000s [97, 98]. **c** Schmidt et al. introduced a microfluidic device in 1990 [99], and the cell encapsulation was studied by Sugiura in 2005 [100]. Zhang et al. generated microgel particles with a capsular structure [101]. Microfluidic emulsification, achieved by Edd et al., offered enhanced controls over a number of encapsulated cells [102]. In 2010, MSC encapsulation was beginning to be studied. **d** The 3D printer was invented by Charles W. Hull in 1983 [103]. The inkjet 3D printing-based hard tissue scaffold was developed by Gima et al. in the early 1990s [104], which was an earlier step for application into soft tissue engineering [105]. Cell-laden and MSC-encapsulated 3D bioprinting was attempted form the 2000s onward after the development of the cell-free printed scaffold [74]. Finally, 4D bioprinting was developed as an advanced bioprinting technique for next-generation technology in the biomedical fields [106]

To overcome the 2D environment, the first attempt of a 3D network for cells was the hanging drop tissue culture system developed by Ross Harrison in 1906 [23]. However, the first introduction of cell encapsulation occurred in the 1960s, when Chang proposed the development of semipermeable microcapsules using nylon membranes by an emulsification method in 1964 [24]. In other studies, using emulsification methods, collagen-agarose [25] and peptides [26] were introduced as the 3D microenvironment for cells. After the early successful strategy by Chang, cell encapsulation within beads and matrices was studied extensively. One of the most common methods for cell encapsulation is entrapment of cells within hydrogel beads which is a very convenient technique that is still being used at present because of its simplicity and stable formation. It was easily achieved by dropping the alginate hydrogel from a syringe into a calcium chloride solution [27]. Aside from the alginate material, this method was also applied to fabricate various cell encapsulation membranes through ionic crosslink formation [28–30]. In addition, processes such as coaxial air jet application and the liquid jet method were developed for cell-entrapped droplet formation. Other developed methods including the macromolecular collagen type I matrix [31], hollow fibers produced by injection and co-extrusion [32], and photopolymerization by UV to reduce cell death without organic solvent were developed. Among these preliminary methods, some technologies are still being used in the same or better way to this day. The widespread and newly developed cell encapsulation technologies, including micromolding, electrostatic droplet extrusion, microfluidics, and bioprinting have been studied since 1958. Recently, microfluidics and bioprinting are widely applied for cell encapsulation. Also, 4D printing technology in which biomaterials or cells are responsive to external stimuli has been developed [33]. With the increasing interest in MSCs, many research groups have started to encapsulate MSCs for cell-based therapy (Fig. 1). Therefore, in this section, we will review the principles behind these technologies with a focus on MSC encapsulation.

### Micromolding method

**Fig. 2:**
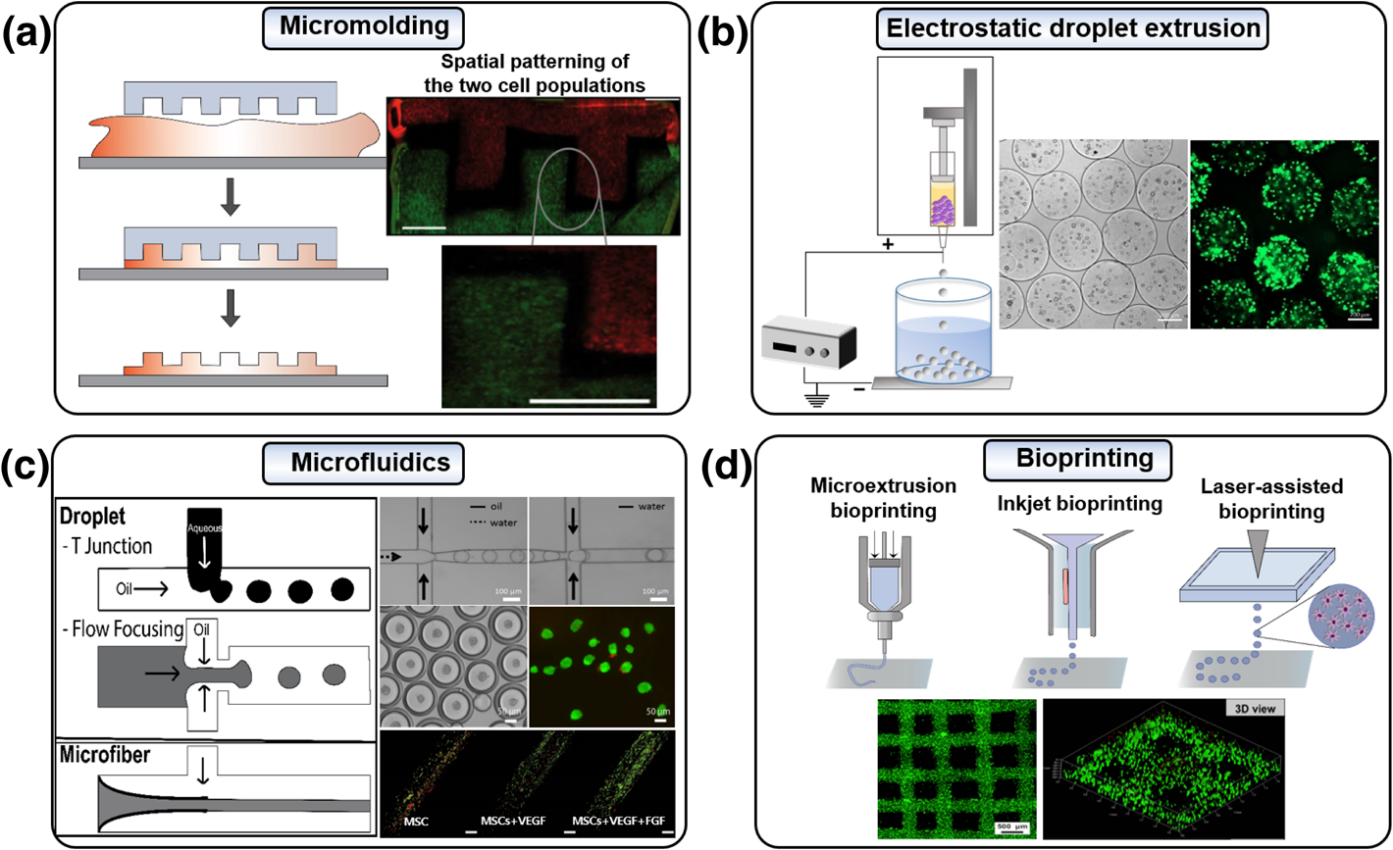
MSC encapsulation technologies. The techniques for encapsulation of MSCs to maintain their viability, proliferation, and differentiation function to deliver the cells into damaged tissues in a 3D microenvironment are achieved through **a** micromolding (reproduced with permission from Reference [38]. Copyright 2013 John Wiley and Sons), **b** electrostatic droplet extrusion, **c** microfluidics (reproduced with permission from Reference [87]. Copyright 2013 Springer Nature and reproduced with permission from Reference [65]. Copyright 2017 IOP Publishing), and **d** bioprinting (reproduced with permission from Reference [107]. Copyright 2018 IOP Publishing) technologies. These technologies create various types of cell encapsulation platforms (e.g., microbeads, bulk matrices, and fiber) and specific shapes

The micromolding method was used to fabricate the cell-laden constructs on a massive scale using specialized lithography equipment. For cell encapsulation, micromolding is a powerful tool that regulates the cellular microenvironment while encapsulating MSCs using specialized lithography equipment (Fig. 2a). Micromolding is the proper technology to manufacture a uniform capsule with excellent reproducibility, which is the reason for its extensive use. There are several types of micromolding methods, including replica, capillary, imprinting, and microtransfer molding. Among these methods, replica molding and capillary molding are easy to manufacture and have excellent repeatability at low cost, so they are currently widely used.

Replica molding is a process in which structures of various shapes and sizes are fabricated by diverse types of molds, using the gelation of a precursor polymer [34]. Polydimethylsiloxane (PDMS) membranes are the most commonly used templates for the gel structures. The polymer solution is poured onto a master made of a silicon substrate and microstructure, and the cured PDMS is then peeled off from the master [35]. To encapsulate cells with molding, the membrane is immersed in a cell-suspended polymer solution and then removed after solidification of the solution. Trkov et al. studied the vasculogenic potential of MSCs through replica molding microfabrication [36]. Vorwald et al. claimed that proper biomaterials should be considered as the molding resource for MSC transplantations; for example, the alginate hydrogel effectively encapsulates MSCs as spheroids [37]. Hamilton et al. designed and implemented a hydrogel replica molding, which successfully cultivated two or more cell types in a highly customized environment that represented the 3D tissue physiology [38]. They have developed a 3D hydrogel co-culture system by separating and isolating different cell populations using enzyme-sensitive glues. This platform enabled co-culture effects and cultivation to test the persistence of paracrine signals and provided great potential for the future study of various basic cell signals.

Micromolding in a capillary is a convenient technique for producing a patterned microstructure of an organic polymer on the surface of a solid substrate. This technology is achieved by flowing a polymer solution (e.g., PDMS fluids) to a prepared capillary patterned mold, whereupon the PDMS adheres tightly to a flat substrate and hardened to create a capillary channel. Then, cell-containing polymer solution is placed at the ends of channels to create cell encapsulating microtube and filled automatically into microchannel by capillary force. The resultant PDMS microchannel creates a partial vacuum inside the cavity and plays a role in attracting the polymer solution [39–41].

### Electrostatic droplet extrusion

As an advanced facile technology relative to the simple syringe extrusion method, the electrostatic droplet extrusion method has been widely studied for MSC microencapsulation (Fig. 2b). Compared with other extrusion techniques such as air jet extrusion and vibrational encapsulation, electrostatic droplet extrusion provides manufactures with smaller beads with a diameter of less than 50 μm [42]. Moreover, the advantage of this technology is that it can be performed under less stressful conditions without the use of organic solvents that reduce cell viability [43]. Even though a high electronic field is loaded for the process, the electrical potential does not affect cell viability because the main principle of electrostatic droplet extrusion is the disruption of a liquid jet by electrostatic forces. In this process, liquid is extruded through a needle connected to the high-voltage generator. Upon application of a potential to the needle, the liquid surface is transformed into a Taylor cone-like droplet, and the liquid cone expands to create a thin strand. At the end of the electrostatic droplet extrusion steps, small polymer droplets are formed by detaching from the aperture of the needle [44]. Furthermore, several factors can affect the size of the droplets, such as the electrostatic potential, the distance between the collecting device and syringe pump, the diameter of the needles, the flow rate, the type of polymer solution used, and the concentrations and viscosity of the solution [45].

As already mentioned above, the biopolymer alginate has been commonly used for cell encapsulation by electrostatic droplet extrusion because of its fast gelation process based on the ionic crosslinking mechanism. To be more specific, alginate is a biodegradable and biocompatible copolymer containing β-d-mannuronic acid (M) and α-l-guluronic acid (G). It has been reported that the G blocks contribute mainly to the gelation of alginate because when divalent cations (e.g., Ca^2+^) are introduced to an alginate solution, the G blocks in the alginate backbone form an ionic inter-chain bridge with the cations [46]. Moreover, the alginate hydrogel has a higher mechanical property than that of other biopolymers and as such is a suitable material for tissue regeneration. In addition, alginate-agarose [47], collagen-terpolymer [48], and arginyl-glycyl-aspartic acid (RGD)-modified alginate microcapsules [49] have been used to encapsulate cells with the electrostatic droplet extrusion technique.

Using this technology, Yao et al. produced alginate microspheres for rat adipose-derived stem cells (rADSCs) [50]. The rADSC-laden microspheres were formed by dropping the cell-suspended alginate solution into a calcium chloride solution. The authors considered four parameters such as voltage, electrode distance, push speed, and an inner diameter of the needle for the encapsulation and transplantation. They found that the critical factor affecting the microencapsulation was the electrode distance, that is the distance between the needle point and the copper sheet. Furthermore, it was shown that increasing the flow rate enhanced the production efficiency. In another study group, Moyer et al. developed human-ADSC (hADSC)-laden microbeads for efficient delivery into the body by percutaneous injection [51]. They encapsulated hADSCs in alginate microbeads using an electrostatic droplet generator and confirmed that smaller beads could be manufactured by increasing the electrical potential to 7 kV and decreasing the flow rate to < 5 mL/h. They proved that at 3 months after percutaneous injection of the encapsulated hADSCs, stable protection of the cells during injection and in the body had been achieved.

### Microfluidic-based cell encapsulation

Microfluidics is a technology based on the manipulation of a fluid in a microscopic environment, such as a microchannel [52]. It involves a variety of microfabrication processes and presents a promising approach toward the rapid and high-throughput containment of cells in microgels [53, 54]. The advantages of this technology are that it enables precise environmental control, uniform size control with low shear stress, and device structure control according to various encapsulation conditions [55]. These microfluidic systems for cell encapsulation can be categorized into two major types: droplets and microfibers (Fig. 2c).

#### Microfluidic encapsulation in droplets

The microfluidic flow-focusing device is applied to cell encapsulation with various junction types. To control the generation of droplets, T-junction and flow-focusing methods can be applied according to the channel geometry [56]. T-junction is coaxial capillary, and micro-nozzle cross-flow system and the size of the orifice of the T-junction greatly affect the size of the droplets formed. In the T-junction method, the main channel of the continuous phase and the inlet channel of the dispersed phase intersect perpendicularly with each other. The two phases form a contact at the junction with continuously flowing fluid in the main channel, and the tip of the dispersed phase enters the main channel. The size of the droplet is changed by controlling the flow rate or channel width, or by varying the relative viscosity [56–58]. In the flow-focusing method, the dispersion and continuous phases are allowed to pass through a narrow region of the microfluidic device. To better control and stabilize the droplets, symmetric shearing is used to place the continuous phase on the dispersed phase. The size of droplets can be reduced by increasing the speed of the fluid in the continuous phase [54, 58, 59]. In hydrogel solutions, cells are generally encapsulated by gelation of the cell suspension to form a coagulated matrix. The gelation depends on the encapsulating material (e.g., gelatin, agarose, alginate, and chitosan) and the cell-laden hydrogel droplets horizontally distributed to the dispersed phase.

Li et al. demonstrated that human bone marrow-derived MSCs (hBMSCs) encapsulated in protein-based microgels can be a therapeutic candidate for the long-term maintenance of articular cartilage regeneration under biocompatible microfluidic processing [60]. Zhao et al. reported a process that utilized this technology to facilitate bone regeneration with minimum invasion [61]. To generate injectable osteogenic tissue constructs, growth factors and rat BMSCs (rBMSCs) were entrapped in photocrosslinkable methacrylated gelatin (GelMA) microspheres. As a result, rBMSCs encapsulated in GelMA microspheres showed enhanced osteogenesis in vitro and in vivo.

#### Microfluidic encapsulation in microfibers

Microfluidic encapsulation in microfibers is defined as the formation of fibers within a microchannel using the coaxial flow of the initiating polymer and crosslinker [62]. Microfluidic fibers can be formed with various polymers, including gelatin-hydroxyphenylpropionic acid, alginate, chitosan, gelatin, and poly (lactic-co-glycolic acid) (PLGA). Microfluidic spinning is the proper technology for cell encapsulation because the fibers can be continuously produced and cover the cells without the need for a high voltage or temperature [63]. In microfluidics, PDMS-based and glass-based microfluidic chips are mainly used. These types of devices are easily extruded using similar materials and methods [63, 64]. Liu et al. reported mouse BMSC encapsulated alginate microfibers using microfluidic technology for vascular grafts [65]. The generated microfibers were controlled by the different flow rates and the diameters of a capillary glass tube.

### Bioprinting

Bioprinting is an emerging technology for tissue regeneration because of its advantages, such as precise control of the cell density, high-resolution cell deposition, controllable scale, and cost-efficiency. The ultimate goal of bioprinting is the production of a living organ on a larger scale for transplantation which is required for many patients. Furthermore, the printed tissue becomes a possible tool for studying cell-cell and cell-matrix interactions in microenvironments by mimicking the 3D extracellular matrix (ECM). The basic concept of this technology is the layered deposition of cell-laden building blocks or cell aggregates using several types of rapid prototyping. There are three primary technologies, namely, inkjet bioprinting, laser-assisted bioprinting, and extrusion bioprinting that each with different processes and benefits (Fig. 2d).

#### Inkjet bioprinting

Inkjet bioprinting was the first technology of bioprinting and has a similar concept as conventional inkjet printing because it uses a cell-bearing pre-polymer solution (called a bioink solution) for ink cartridge dispensing [66]. The bioink is squeezed to produce bioink droplets through stimulation of the printer heads by a thermal piezoelectric activator [67]. Despite its low cost and high-throughput production of the parallel cell arrays, it is limited as a proper technology for tissue regeneration because it can only generate 2D tissue structure. Moreover, the disadvantages of nozzle clogging when used with high cell densities, non-uniform droplet size, lack of structural integrity between droplets, and the possibility of cell exposure to high heating temperature or mechanical stress are substantial challenges of this technology [68].

Although inkjet bioprinting fabricates a 2D structure, the attempt to generate 3D tissue constructs through a layer-by-layer method has been widely studied by many research groups. Gao et al. developed a highly mechanical bone and cartilage using inkjet printing with hMSCs [69]. They generated a bioprinted PEG-GelMA composite scaffold layer by layer to introduce a 3D structure for embedding hMSCs. They confirmed the osteogenesis and chondrogenesis of the hMSCs for 21 days, with a high compressive modulus ranging from 1 to 2 MPa. Another research group studied the differentiation ability of hMSCs in hydrogel blends of type 1 collagen- and chitosan-agarose that were printed by inkjet bioprinting [70]. The single drops of hydrogel were dispensed on top of one another to form a solid column. The hMSCs showed differentiation tendency toward osteogenic and adipogenic lineages, confirming that an anisotropic soft collagen-enriched 3D matrix was suitable for adipogenic differentiation, whereas an agarose-enriched matrix induced osteogenic differentiation. Therefore, these results showed that a 3D matrix printed by inkjet bioprinting was suitable for guiding MSC differentiation fates, and different types of tissue can be realized through control of the matrix stiffness.

#### Extrusion bioprinting

The most common type of 3D bioprinting is extrusion bioprinting, which was developed from the inkjet bioprinting technology. It involves extrusion printers, such as pneumatic and mechanical (piston or screw) dispensing systems, to extrude bioink droplets sequentially by forming cylindrical lines under a steady force. The major advantage of extrusion bioprinting is the ability to dispense bioinks with high cell density, which is essential for forming 3D tissue-derived organs [71]. Furthermore, since a wide range of viscous materials can be used, the selection of a variety of materials to encapsulate the cells is possible. Extrusion bioprinting is also able to produce multicellular spheroids for forming self-assembled tissue and organ constructs [72].

Using a customized microextrusion bioprinter, Du et al. fabricated a 3D bioprinted structure of rBMSC-laden GelMA scaffolds in the micrometer scale with a collagen-binding domain [73]. They confirmed the high cell viability (> 90%) and osteogenic differentiation of BMSCs for 14 days in the osteogenic medium by evaluating the gene expression of osteogenic markers, such as alkaline phosphatase (ALP), bone sialoprotein, osteocalcin (OCN), and collagen type I. Another study group Levato et al. also established bone constructs from MSC-laden poly(lactic acid) microcarriers encapsulated in a GelMA-based hydrogel through extrusion bioprinting [74]. However, there is an obstacle of decreasing cell viability because of the extrusion pressure and the small diameter of the nozzle [75]. Therefore, enhancement of the cell viability and maintenance of the cell function in bioprinting composites are critical challenges for extrusion bioprinting technology.

#### Laser-assisted bioprinting

Laser-assisted bioprinting, which is based on laser-induced transfer, consists mainly of three components: a pulsed laser source, a donor layer, and a receiving substrate [76]. The donor layer is composed of an energy-absorbing layer (e.g., gold or titanium) on the top and a layer of mixed bioink solution (e.g., hydrogel and/or cells) on the bottom. A high-pressure bubble is formed at the interface of the bioink layer after the energy-absorbing layer is stimulated by focused laser pulses which evaporate donor layer part, and then the cell-containing solution is propelled to the receiving substrate [77]. The advantage of this technology is the high cell viability achieved as a result of the low mechanical stress on the cells due to the non-direct method between the bioink and dispenser. Furthermore, it is possible to deposit highly viscous materials with the high cell density concentrated bioink [78], and the bioprinter is able to use various types of bioink.

Gaebel et al. used modified laser-assisted bioprinting to develop a polyester urethane urea cardiac patch seeded with hBMSCs and HUVECs in a controlled pattern for cardiac regeneration [79]. They demonstrated that the co-printing of hBMSCs and HUVECs in the cardiac patch enhanced both the angiogenesis and the functional neo-vasculature that improves the functionality of an infarcted heart after transplantation. In another study group, Gruene et al. generated porcine BMSC-embedded 3D constructs by laser-induced forward transfer to confirm the viability and functionality of cells and their differentiation into bone and cartilage phenotypic cells [80]. They verified that laser-assisted bioprinting was capable of printing with a high cell density, which is essential for 3D tissue formation, and maintained cell viability during the printing process. Moreover, the MSC differentiation ability was observed from the expression of the osteogenic markers OCN and ALP and the chondrogenic markers collagen type II and aggrecan.

## Applications of cell encapsulation as regenerative medicine

Cell encapsulation technologies provide a biomimetic 3D environment for MSCs to maintain their viability and functionality, resulting in their differentiation into many types of tissue. The spatiotemporal 3D environment affects MSC fate by matrix composition, substrate stiffness, porosity, and substrate structure. The different 3D environments influence integrin interaction and clustering between the MSCs and matrix which have cell adhesion and proliferation. These affect the signaling of the cell, that is transduced to the nucleus to regulate gene expression and consequently to alter the cell phenotype [81]. Therefore, tissue regeneration requires various environments depending on a tissue type, because of their distinct ECM parameters, respectively. In addition, biomechanical stimuli are not enough to enhance MSC differentiation, so the combination with biochemical components such as growth factors including BMP-2, TGF- β1, VEGF, and FGF is critical [82]. 3D encapsulation technologies can provide physicochemical effects on MSCs by encapsulating both MSCs and growth factors. Thus, MSC encapsulation technologies that can provide diverse shapes, sizes, and matrix compositions with biochemical cues must be optimized depending on a targeted tissue. Among the various types of tissue, cartilage and bone are representatives of well-studied hard tissues. Moreover, the skin is a normal soft tissue that has been extensively studied. Blood vessel regeneration is also an important consideration because it is an essential requirement for all tissue regenerations. In this section, we will discuss the use of the MSC encapsulation technologies described above for the regeneration of these four types of tissue.

### Cartilage

**Fig. 3:**
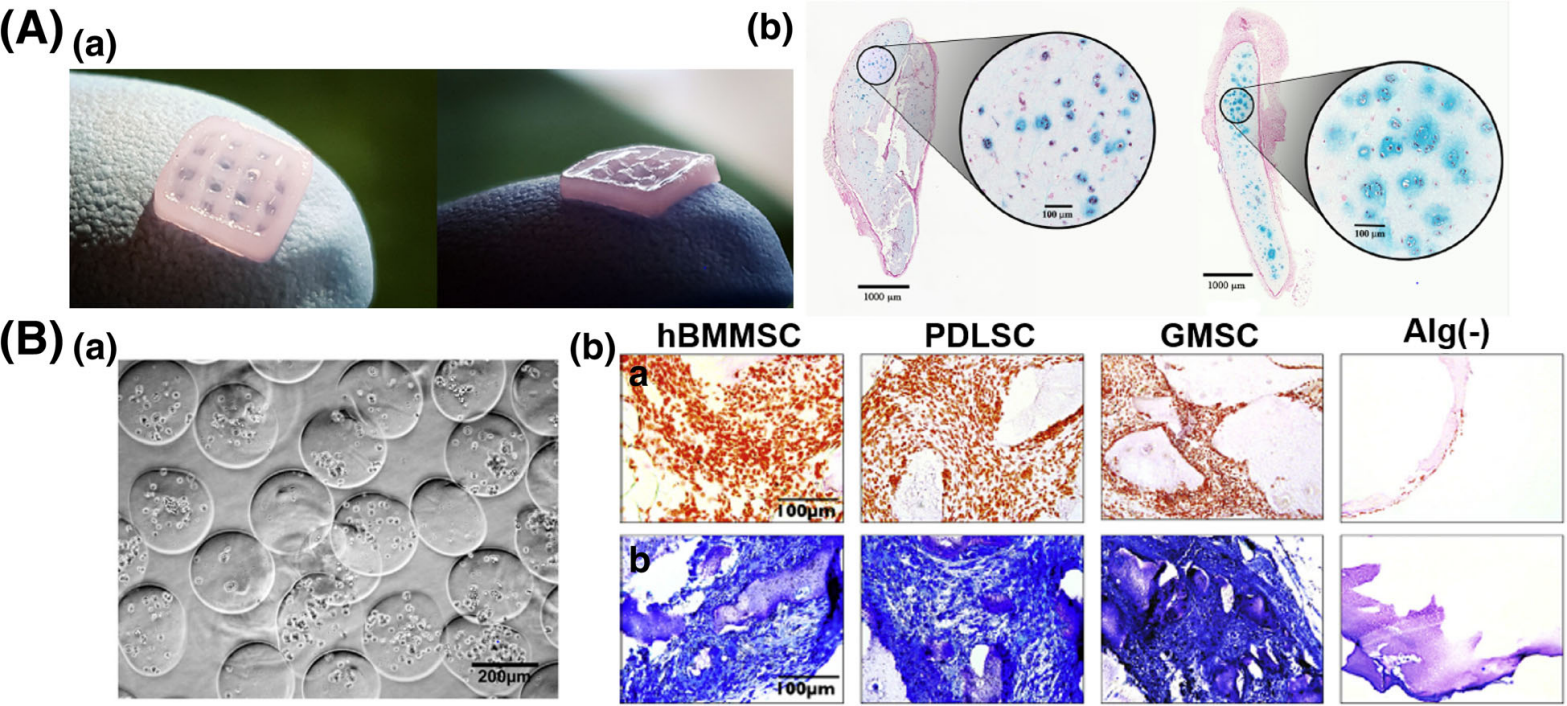
Application of MSC encapsulation for cartilage regeneration. **A** (*a*) Cell-encapsulated nanofibrillated cellulose bioprinting gel. (*b*) Chondrocyte proliferation in 3D-bioprinted scaffold with hNCs and hBMSCs at day 30 (left) and day 60 (right) after subcutaneous implantation. Reproduced with permission from Reference [83]. Copyright 2017 PLOS. **B** (*a*) The periodontal ligament stem cells (PDLSCs), gingival mesenchymal stem cells (GMSCs), and hBMSC-encapsulated RGD-coupled alginate microbeads with TGF-β1 by microfluidic device. (*b*) MSC-encapsulated microbeads stained safranin-O and toluidine blue that indicates proteoglycans, significantly. Reproduced with permission from Reference [84]. Copyright 2013 Elsevier

Injured cartilage is one of the most difficult tissues to regenerate because it is one of the avascular tissues with a limited supply of oxygen and nutrients. As a conventional treatment, autologous chondrocyte cell therapy has been widely applied, but it has drawbacks, such as its complexity, loss of cell functionality, and incomplete healing. Therefore, the method of MSC delivery to the defective site to induce differentiation toward chondrocytes is an emerging therapy for cartilage regeneration. Many research groups have examined various types of 3D environment scaffolds for delivering MSCs and inducing chondrogenesis. Peter et al. used extrusion bioprinting to encapsulate hBMSCs and human nasal chondrocytes creating human cartilage in vivo (Fig. 3A) [83]. Encapsulated hBMSCs enhanced the proliferation of chondrocytes and the formation of cartilage clusters. In addition, using a microfluidic device, Alireza et al. established a co-delivery system of TGF-β1 and human dental MSCs (hDMSCs) in RGD-coupled alginate microspheres (Fig. 3B) [84]. In their study, the hDMSCs (2 × 10^6^/ml in alginate) differentiated into chondrocytes upon the presentation of TGF-β1 as an inductive signal. The alginate microsphere-using microfluidic device was simple and maintained MSC viability and chondrogenic differentiation properly.

### Bone

**Fig. 4:**
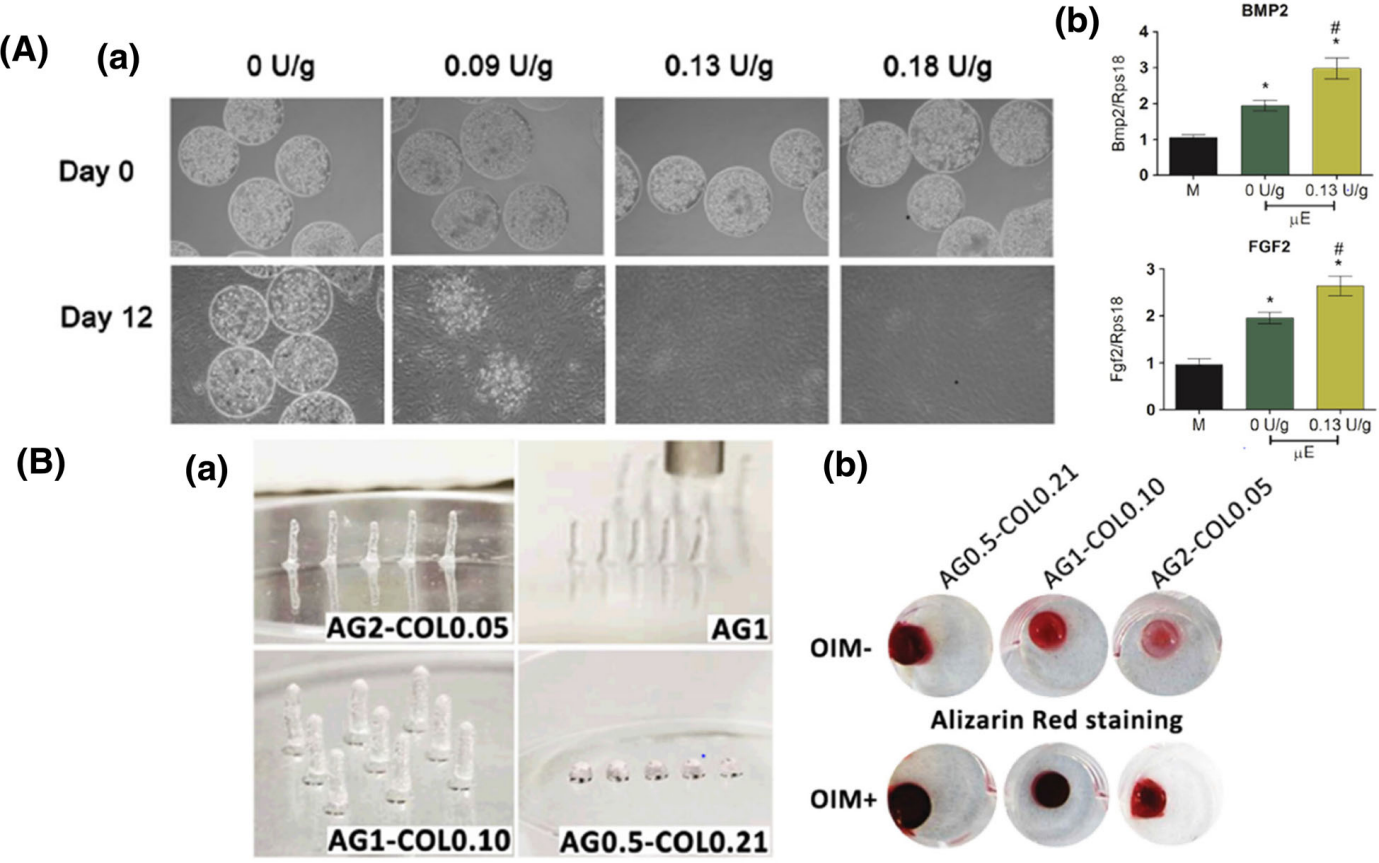
Effective MSCs delivery for bone regeneration. **A** (*a*) Release of ADSCs from alginate microbeads with different concentration of alginate-lyase by electrostatic droplet extrusion. (*b*) ADSC-encapsulated alginate-lyase microbeads revealed high expression of BMP-2 and FGF-2 that regulates bone regeneration. Reproduced with permission from Reference [85]. Copyright 2013 Elsevier. **B** (*a*) Printed agarose-collagen 3D columns and rings using inkjet bioprinting. (*b*) Alizarin red staining for hBMSC-loaded agarose-collagen hydrogel scaffold. Reproduced with permission from Reference [86]. Copyright 2016 John Wiley and Sons

The implantation of a cell-laden 3D scaffold is important for the induction of osteogenesis in MSCs and the reconstruction of bone tissue. Leslie et al. encapsulated rADSCs in alginate and alginate-lyase combination hydrogel microbeads (< 200 μm) at a concentration of 25 × 10^6^ cells/ml using an electrostatic droplet generator (Fig. 4A) [85]. They controlled the degradation rate via the ratio of alginate-lyase to alginate for maintaining rADSCs viability in the injured site. The rADSCs released from the alginate-lyase microbeads were still viable after 12 days, and they released a high level of bone morphogenetic protein 2, VEGF-A, and FGF2, which are factors that stimulate bone regeneration. In another study group, Daniela et al. encapsulated hBMSCs in 3D agarose-collagen scaffold using inkjet bioprinting [86]. They confirmed that agarose is important to increase bioprinting contour accuracy, and collagen is crucial to enhance hBMSC proliferation resulting in osteogenic differentiation. As a result, high collagen concentration of scaffold was effective for bone regeneration. Chan et al. studied assembled cell spheroids by application of the microfluidic-based water-in-oil-in-water double-emulsion method [87]. The aggregated hBMSCs formed spheroids in the alginate and RGD-modified alginate droplets using 8 × 10^6^ cells/ml. Furthermore, the hBMSC spheroids in the RGD-modified alginate showed enhanced osteogenic differentiation after 7 days in culture. The successful bone regeneration was clarified by alizarin red staining and an increase in ALP activity (Fig. 4B).

### Skin

**Fig. 5:**
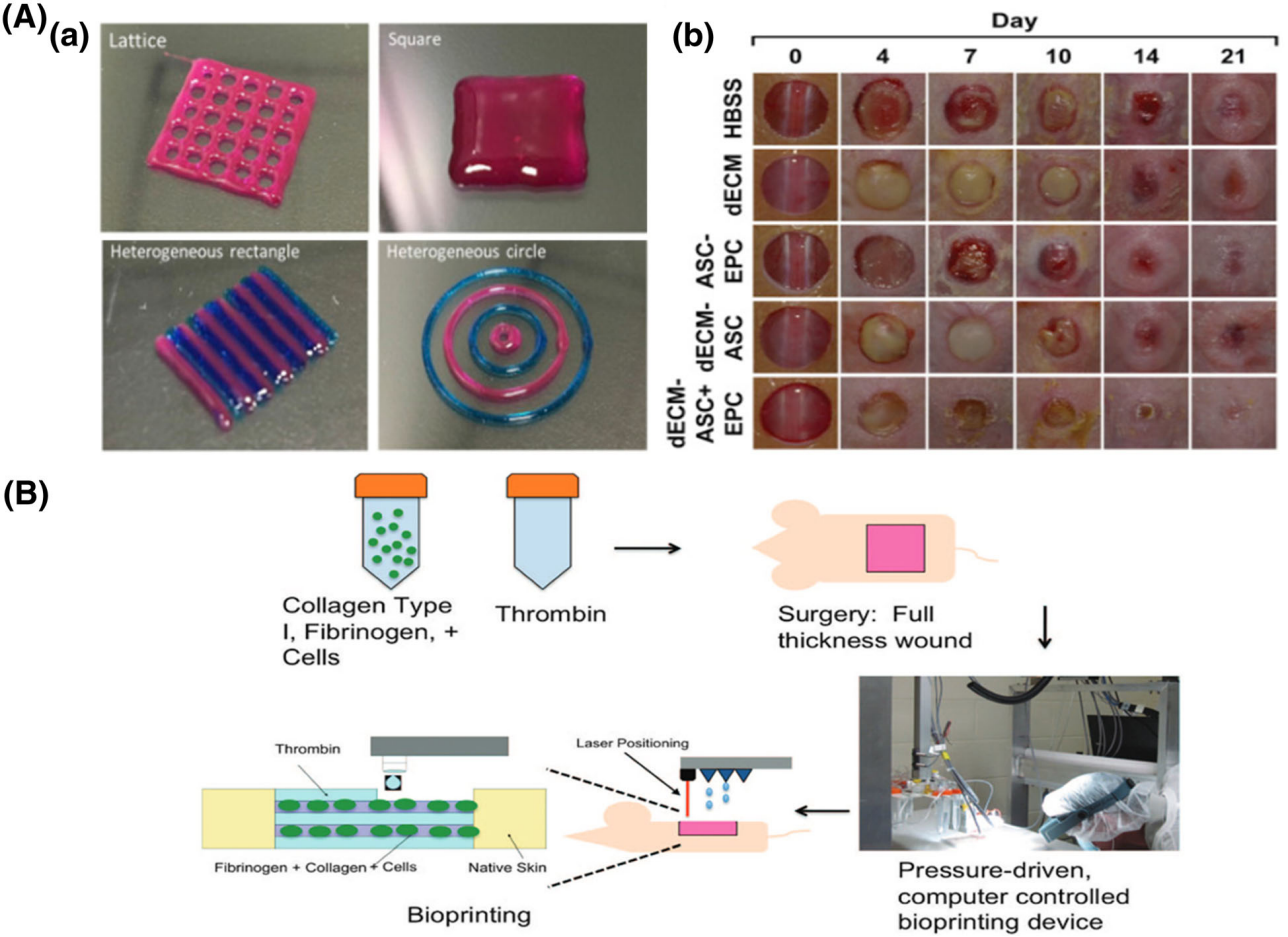
MSC encapsulation using bioprinting for treatment of skin regeneration. **A** (*a*) Printability test of dECM bioink through the production of heterogeneous structure by modeling. (Bioink A: cell-free S-dECM bioink was stained with rhodamine (red); bioink B: cell-free S-dECM bioink was stained with trypan blue(blue). (*b*) The images of wound healing for 21 days. Reproduced with permission from Reference [89]. Copyright 2018 Elsevier. **B** Schematic diagram illustrating an approach to bioprinting amniotic fluid-derived stem (AFS) cells to increase the healing of full-thickness skin wounds. Reproduced with permission from Reference [90]. Copyright 2012 John Wiley and Sons

There has been a lot of research to reconstruct damaged skin, which covers most of the human body. Generally, burned tissue and chronic wounds have been successfully treated with scaffold-based tissue constructs [88]. Kim et al. introduced a skin-derived ECM bioink to fabricate a 3D thickness skin construct by bioprinting [89]. They made a 3D-printed skin patch with hADSCs and endothelial progenitor cells for wound healing. In vivo results demonstrated that the hADSCs enhanced wound healing, re-epithelialization, and the formation of new blood vessels that realize skin regeneration using 6 × 10^6^ cells/ml in media (Fig. 5A). Skardal et al. also used bioprinting technology to form full-thickness skin tissue on mice wounds [90]. hBMSCs and human amniotic fluid MSCs (hAFMSCs) that were suspended separately in a fibrin-collagen gel were printed over the wound site of mice. The hBMSC- and hAFMSC (5 × 10^6^ cells/scaffold)-encapsulated scaffolds accelerated wound closure and re-epithelialization more significantly than did a cell-free fibrin-collagen scaffold. They concluded that growth factors secreted by hAFMSCs had induced angiogenesis and wound closure at the injured site (Fig. 5B).

### Blood vessel

**Fig. 6:**
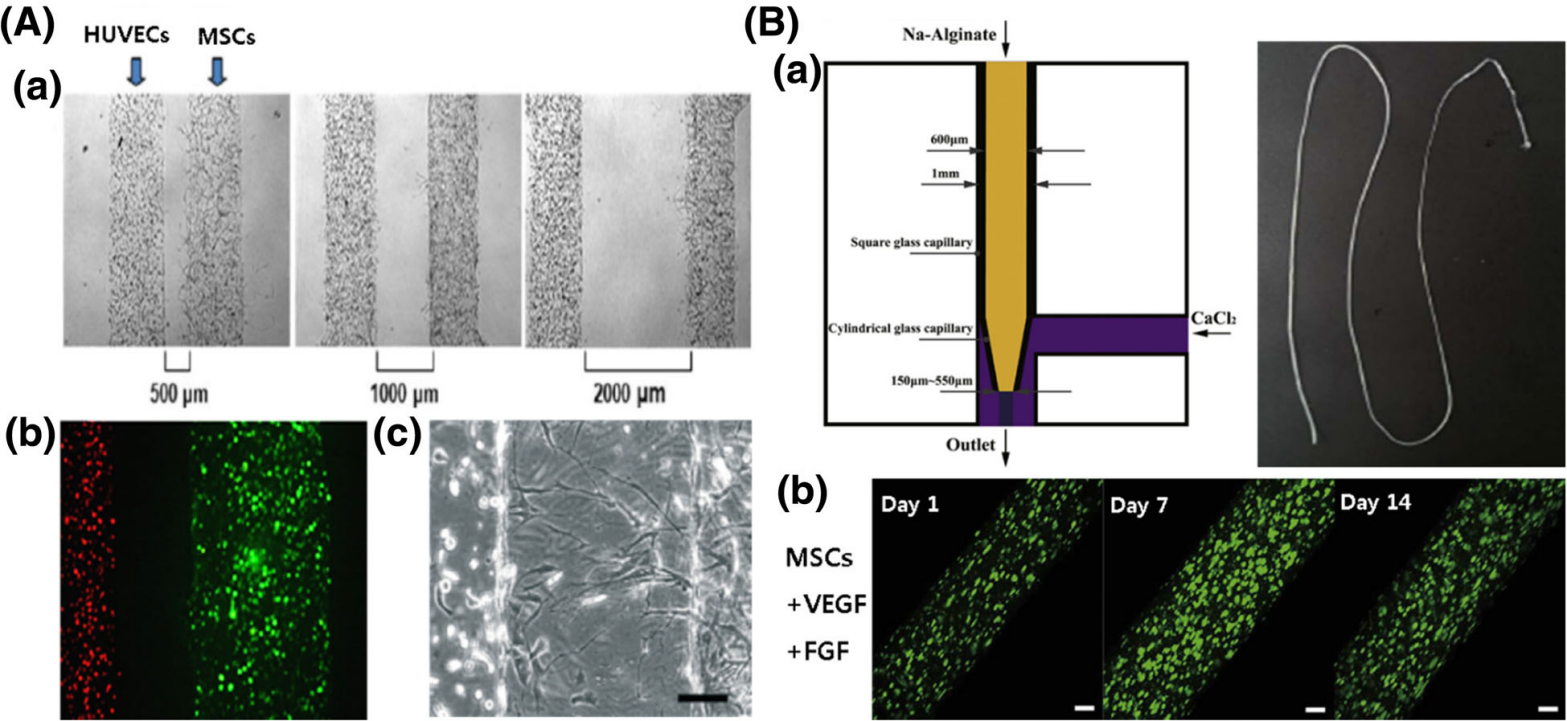
Blood vessel regeneration by MSC encapsulated 3D construction. **A** (*a*) The 3D co-culture system of mesenchymal and endothelial cells in the micropatterned hydrogel. HUVEC- and MSC-loaded micropatterned fibrin channels with the distances between channels of 500, 1000, and 2000 μm. (*b*) Encapsulated HUVECs (left channel: red) and MSCs (right channel: green). (*c*) MSCs sprouted to HUVEC with distance-dependent response (scale bar,100 mm). Reproduced with permission from Reference [36]. Copyright 2009 John Wiley and Sons. **B** (*a*) Schematic diagram of microfiber generation and principle of gelation and actual shape. (*b*) MSCs with VEGF and FGF were effective for angiogenesis in microfiber over 14 days. Scale bar, 200 μm. Reproduced with permission from Reference [65]. Copyright 2017 IOP Publishing

The reconstruction of blood vessels plays a key role in tissue regeneration because they supply oxygen and nutrients to tissues. As the source for regenerating blood vessels, MSCs are good candidates considering their unique antithrombogenic properties, immune response, and multipotency to differentiate into vascular phenotypes. Trkov et al. emphasized that unraveling the mechanism of blood vessel formation would offer therapeutic solutions because there are many limitations in oxygen and nutrient supply [36]. HUVEC- and hBMSC-encapsulated hydrogels were localized for each channel using microfluidic patterning (10^6^ cells/100 μl in PBS). The authors used a simple and robust mold to study communication within the cells of relevance for vascularized tissue engineering (Fig. 6A). Liu et al. investigated small diameter of vascular grafts through microfiber using microfluidic device. Encapsulated mouse BMSCs proliferated in the microfibers with VEGF and fibroblast growth factor (FGF) showed stable vascular regeneration (Fig. 6B) [65]. Jeon et al. described a number of factors related to vessel development, and the non-endothelial cell-specific growth factors, such as the proteins of the TGF family that are also required for angiogenesis [91]. They demonstrated that two endothelial cell-related molecules, angiopoietin and TGF-β1, released from encapsulated hBMSCs (6 × 10^6^ cells/ml in hydrogel suspension) using a microfluidic device, played an important role in angiogenesis.

## Conclusion

Tissue regeneration is one of the most prominent and vital approaches in diverse biomedical applications. MSCs, which are known to have a lot of potential and play an increasingly leading role in tissue engineering and regenerative medicine, have only recently been applied as a biomedicine. In this review, we have investigated various MSC encapsulation technologies and their applications in tissue regeneration. For clinical applications, we need to use a variety of techniques, but among them, we have looked at four technologies: micromolding, electrostatic droplet extrusion, microfluidics, and bioprinting. The advantages of MSC encapsulation technologies are improvement of MSC viability, proliferation, differentiation capacity, and protection of MSCs from the immune system in the body. These technologies which are composed using 3D environment structure can provide safe delivery of MSCs into a specific site of defection, which can provide long-term cell-based therapy for tissue regeneration. Therefore, mass production of MSCs and scaled-up matrix are critical challenges that are to be considered for clinical application of the tissue regeneration. In other words, cost, time, and labor efficiency of cell encapsulation and transplantation are challenges. Additional challenge is to consider the encapsulation biomaterial as the immune response to the implanted MSC-encapsulated matrix. Thus, the simple and easy technique to encapsulate MSCs and a combination of biocompatible materials must be improved for effective tissue regeneration.

Previous tissue engineering methods using MSC encapsulation have used each technology of micromolding, electrostatic droplet extrusion, microfluidics, and bioprinting individually. However, we need to combine these technologies to overcome the limitations of each and thereby increase their advantages. Besides this, although MSCs are considered to be an attractive cell source owing to their multipotency, it is difficult to ignore their possible differentiation into an abnormal tissue. Through a preliminary study of the differentiation of MSCs and their effects, diseases caused by unexpected differentiation can be prevented and treated. Therefore, MSC encapsulation technology has the potential to be utilized infinitely in therapeutic medicine by suppressing the abnormal differentiation and side effects of MSCs. In conclusion, MSC encapsulation is a very promising technology for use in tissue engineering and regenerative medicine applications, depending on the occasion and purpose.

## Abbreviations

2D: Two-dimensional
3D: Three-dimensional
ALP: Alkaline phosphatase
ECM: Extracellular matrix
FGF: Fibroblast growth factor
G: α-l-Guluronic acid
GelMA: Methacrylated gelatin
hADSCs: Human adipose-derived stem cells
hAFMSCs: Human amniotic fluid MSCs
hBMSCs: Human bone marrow-derived MSCs
hDMSCs: Human dental MSCs
hNCs: Human nasal chondrocytes
HSPCs: Hematopoietic stem/progenitor cells
HUVECs: Human umbilical vein endothelial cells
IL-2: Interleukin-2
IL-8: Interleukin-8
M: Containing β-d-mannuronic acid
MCP-1: Monocyte chemotactic protein-1
MSC: Mesenchymal stem cell
OCN: Osteocalcin
PDMS: Polydimethylsiloxane
PLGA: Poly (lactic-co-glycolic acid
rADSCs: Rat adipose-derived stem cells
rBMSCs: Rat bone marrow-derived MSCs
RGD: Arginyl-glycyl-aspartic acid
SDF-1: Stromal-derived factor-1
TGF-β: Transforming growth factor-beta
VEGF: Vascular endothelial growth factor
